# Taxonomic novelties and host associations of *Sordariomycetes* from wetland-dwelling *Poales* in Thailand

**DOI:** 10.3897/mycokeys.129.163092

**Published:** 2026-03-16

**Authors:** Amuhenage T. Bhagya, Chayanard Phukhamsakda, Pattana Kakumyan, Ali H. Bahkali, E. B. Gareth Jones, Kevin D. Hyde

**Affiliations:** 1 Center of Excellence in Fungal Research, Mae Fah Luang University, Chiang Rai 57100, Thailand Center of Excellence in Fungal Research, Mae Fah Luang University Chiang Rai Thailand; 2 School of Science, Mae Fah Luang University, Chiang Rai 57100, Thailand School of Science, Mae Fah Luang University Chiang Rai Thailand; 3 Department Microbial Drugs, Helmholtz Centre for Infection Research (HZI), Inhoffenstrasse 7, 38124, Braunschweig, Germany College of Science, King Saud University Riyadh Saudi Arabia; 4 Department of Botany and Microbiology, College of Science, King Saud University, P.O Box 2455, Riyadh, 11451, Saudi Arabia Department Microbial Drugs, Helmholtz Centre for Infection Research (HZI) Braunschweig Germany

**Keywords:** 2 new species, 2 new host records, aquatic fungi, fungal taxonomy, *
Naviculisporaceae
*, *
Podosporaceae
*

## Abstract

*Poales* is one of the largest orders of monocotyledonous plants, comprising families such as *Cyperaceae*, *Poaceae*, and *Typhaceae*. These plant families dominate the macrophytes found in tropical lentic and lotic wetlands habitat in Prachuap Khiri Khan Thailand. This study investigated microfungi associated with wetland-dwelling *Poales* and revealed taxa belonging to *Chaetomiaceae*, *Naviculisporaceae*, *Pleurotheciaceae*, and *Schizotheciaceae* in *Sordariomycetes* were prominent. Submerged and decaying plant material from *Cyperaceae* and *Typhaceae* were gathered from the Pran Buri marshlands and the Pran Buri River, Thailand. The spatial distribution of the locations was significantly influenced by fluctuations in ocean water levels. Fungal characterization was performed using morphological analysis and multigene phylogeny, incorporating nuclear ribosomal DNA (ITS, LSU, SSU) and protein-coding loci (*rpb2*, *tub2*, *tef1-α*). This study introduces two novel species, *Corynascus fluvialis* and *Pseudorhypophila hyalibasiconica* from *Carex* sp. along with two new host records: *Dematipyriforma aquilariae* from *Typha* sp. and *Triangularia allahabadensis* from *Carex* sp. *Pseudorhypophila hyalibasiconica* is characterized by ovoid to limoniform upper cells and a hyaline conical lower cell. *Corynascus fluvialis* differs from other members of *Corynascus* by possessing angular pedicellate asci, and ascospores with a single apical germ pore. The comparisons using morphology and phylogeny of newly described taxa are provided. This study investigates the role of sustainably managed forests in the conservation of terrestrial ecosystems.

## Introduction

*Sordariomycetes* is the second largest class of *Ascomycota*, with a cosmopolitan distribution and variety of life modes including saprobic, pathogenic, and endophytic. While the majority of *Sordariomycetes* species inhabit terrestrial environments, a significant number have also been reported from aquatic ecosystems (Hyde and Lee 1998; Calabon et al. 2022). Hyde et al. (2024) listed 45 orders, 167 families and 1,499 genera directly under the class *Sordariomycetes* with 308 genera with uncertain placement in the class. Fungi in this class are characterized by non-lichenized, perithecial ascomata and inoperculate unitunicate asci.

Aquatic fungi under the class *Sordariomycetes* have important adaptations that facilitate their life associated with aquatic environments or directly lead to life in water (Hyde and Lee 1998; Pinruan et al. 2002). The lignocellulosic enzymes produced by fungi in the community help with organic degradation and fulfil their nutritional requirements from aquatic litter (Bucher et al. 2004; Luo et al. 2019). Unique morphological features of aquatic *Sordariomycetes* include well-developed apical rings in the asci, which assist in dispersing ascospores for greater distances (Calabon et al. 2023). Ascospores and conidia produced by aquatic *Sordariomycetes* frequently bear appendages or hyaline cells at the ends (Hyde and Jones 1988; Hyde and Lee 1998). The development of unique characters for the spores help adherence to substrates in lentic water or enable them to float in water columns to colonize new areas (Pang et al. 2013; Luo et al. 2019; Calabon et al. 2023). For example, Pang et al. (2013) highlighted studies on the *Lignincola laevis* complex within *Halosphaeriaceae*, along with the geographical distribution of its strains, providing evidence that suggests the potential for spore dispersal through ocean currents.

Approximately 7.5% of the available land surface of Thailand is covered by wetland ecosystems (Trisurat 2006), include the research sites of this study, such as Khao Sam Roi Yot area and Pran Buri wetland. The economy of local communities surrounding those wetlands, heavily depends on the resources provided by this habitat. The sustainability, conservation, and longevity of wetlands have a significant impact on both the biodiversity and economic stability of Thailand. Fungal communities are vital for ecosystem health (Trisurat 2006), in ensuring nutrient cycling and a balance between species that occupy wetland ecosystems (Samaradiwakara et al. 2023). This study reports the discovery of two new species and two new host records belonging to the class *Sordariomycetes*, isolated from coastal wetland ecosystems in Thailand. These findings contribute to a broader understanding of the fungal communities inhabiting wetland ecosystems in Thailand.

## Materials and methods

### Sample collection, morphological studies, and isolation

Dead and decomposing plant materials belonging to the family *Cyperaceae* and *Typhaceae* were collected from Pranburi wetland and Parnburi River in Prachuap Khiri Khan Province, Thailand. Samples were segregated into each family and packed into air-tight zip-lock bags and transferred to the laboratory. Morphological observations were conducted based on Senanayake et al. (2020), using an OLYMPUS SZXI6 stereomicroscope (Olympus Corporation, Japan), and a Nikon ECLIPSE Ni compound microscope (Nikon Instruments Inc., Japan). The important features were photographed with a Nikon DS-Ri2 digital camera fitted to the microscope. Morphological features of fungi were organized into photo-plates by using Adobe Photoshop CS3 Extended version 10.0 software (Adobe Inc., California, USA). Measurements were taken by utilizing Image Framework software (Tarosoft, Nonthaburi, Thailand). Single spore isolation technique was used to obtain axenic cultures and each culture was grown in Malt Extract Agar (MEA) for 4 to 6 weeks at room temperature (25 ± 5 °C) prior to photography of the culture characteristics or obtain morphological features from culture. Holotype material was deposited in Mae Fah Luang University Herbarium (MFLU) and ex-type living cultures deposited at Mae Fah Luang University culture collection (MFLUCC). Faces of fungi numbers and Index Fungorum numbers were obtained by following Jayasiri et al. (2015) and Index Fungorum (2025) (accessed 15^th^ August 2025). Data on the taxa will be deposited in the Greater Mekong Subregion database (Chaiwan et al. 2021).

### DNA extraction, PCR amplification and sequencing

Seven day to ten days old cultures developed on MEA were used to extract genomic DNA using the E.Z.N.A Fungal DNA Mini Kit-D3390-02 (Omega Bio-Tek, USA) and the manufacturer’s instructions followed. The purified DNA was stored at 4 °C and -20 °C for short and long-term storage. Polymerase chain reactions (PCR) reactions were used for amplification of selected loci (Table 1). The PCR mixture was made to obtain 25 µl as the final volume (12.5 μl of 2× Power Taq PCR master mix, 9.5 µl of deionized H_2_O, 1 µl from each primer at 20 mm/µl concentration, and 1 µl of genomic DNA at 15 ng/µl concentration). Selected loci were amplified using primers and PCR conditions as listed in Table 1. Safe view DNA stain (SOMBIO Taiwan, China) was applied to stain the PCR products, and 1.7% agarose gel electrophoresis was used to separate PCR products. Gel visualization to observe the bands was conducted with the aid of an E-Box CX5 gel documentation system (Vilber Lourmat Deutschland GmbH, Eberhardzell, Germany), and sequencing was done by Solgent Corporation, Yuseong-gu, Daejeon, Korea.

**Table 1.** The amplified loci, primers, and PCR thermal cycle protocols used in the study.

Loci	PCR primers (forward/reverse)	PCR conditions	Reference
**LSU**	LR0R/LR5	95 °C; 5 min (94 °C; 30 s, 55 °C; 50 s, 72 °C; 90 s) × 35 thermal cycles, 72 °C; 10 min	Vilgalys and Hester (1990)
**SSU**	NS1/NS4		
**ITS**	ITS1/ITS4	94 °C; 2 min (95 °C; 30 s, 55 °C; 50 s, 72 °C; 90 s) × 35 thermal cycles, 72 °C; 10 min	White et al. (1990)
***tub2***	Bt2A/Bt2B	95 °C; 5 min (95 °C; 30 s, 58 °C; 90 s, 72 °C; 60 s) × 40 thermal cycles, 72 °C; 10 min	Cai et al. (2006)
***tef1-α***	EF1-983f/EF1-2218r	95 °C; 5 min (95 °C; 30 s, 52 °C; 90 s, 72 °C; 60 s) × 40 thermal cycles, 72 °C; 10 min	Cai et al. (2006)
***rpb2***	fRPB2-5f/	96 °C; 5 min (94 °C; 30 s, 52 °C; 30 s, 72 °C; 60 s) × 40 thermal cycles, 72 °C; 10 min	Cai et al. (2006)
	fRPB2-7cR		

### Phylogenetic analyses

The quality of the raw data was checked by BioEdit v 7.0.9.0 software (Hall 1999) and Lasergene SeqMan Pro v.7 (DNASTAR, Inc, USA) used to generate consensus sequences (Afshari et al. 2023). BLASTn searches at NCBI (https://blast.ncbi.nlm.nih.gov/Blast.cgi) and related literature were utilized to find highly similar sequences (Cai et al. 2006; Phookamsak et al. 2019). All the sequences used in each phylogenetic analysis were aligned in MAFFT 6.864b online tool with the FFT-NS-i method (Katoh et al. 2019) and trimmed manually by BioEdit v 7.0.9.0 software. Low quality sections, primer binding sites and those that were ambiguously based were manually removed from the alignment. Each aligned data set were individually subjected to maximum likelihood (ML) and Bayesian inference (BI) analyses. The IQ-Tree web server (http://iqtree.cibiv.univie.ac.at/) with 1,000 pseudoreplicates was used for ML tree construction and MrBayes v. 3.2.7a (Ronquist et al. 2012) on XSEDE in CIPRES Science Gateway (Miller et al. 2010) was used for BI analysis. Optimum models for each locus were selected through jModelTest2 in CIPRESS. Figtree version 1.4.4 (http://tree.bio.ed.ac.uk/software/figtree/) was used to visualize phylogenetic trees and edited in Microsoft PowerPoint, Microsoft Corporation, Washington, United States.

## Results

### Phylogenetic analysis

Phylogenetic evidence, together with morphological characteristics, confirmed the taxonomic placements of each taxon using a polyphasic approach. The multilocus phylogenetic analysis for *Corynascus*, *Dematipyriforma*, *Pseudorhypophila* with *Triangularia* were conducted using related available strains.

**Figure 1.**
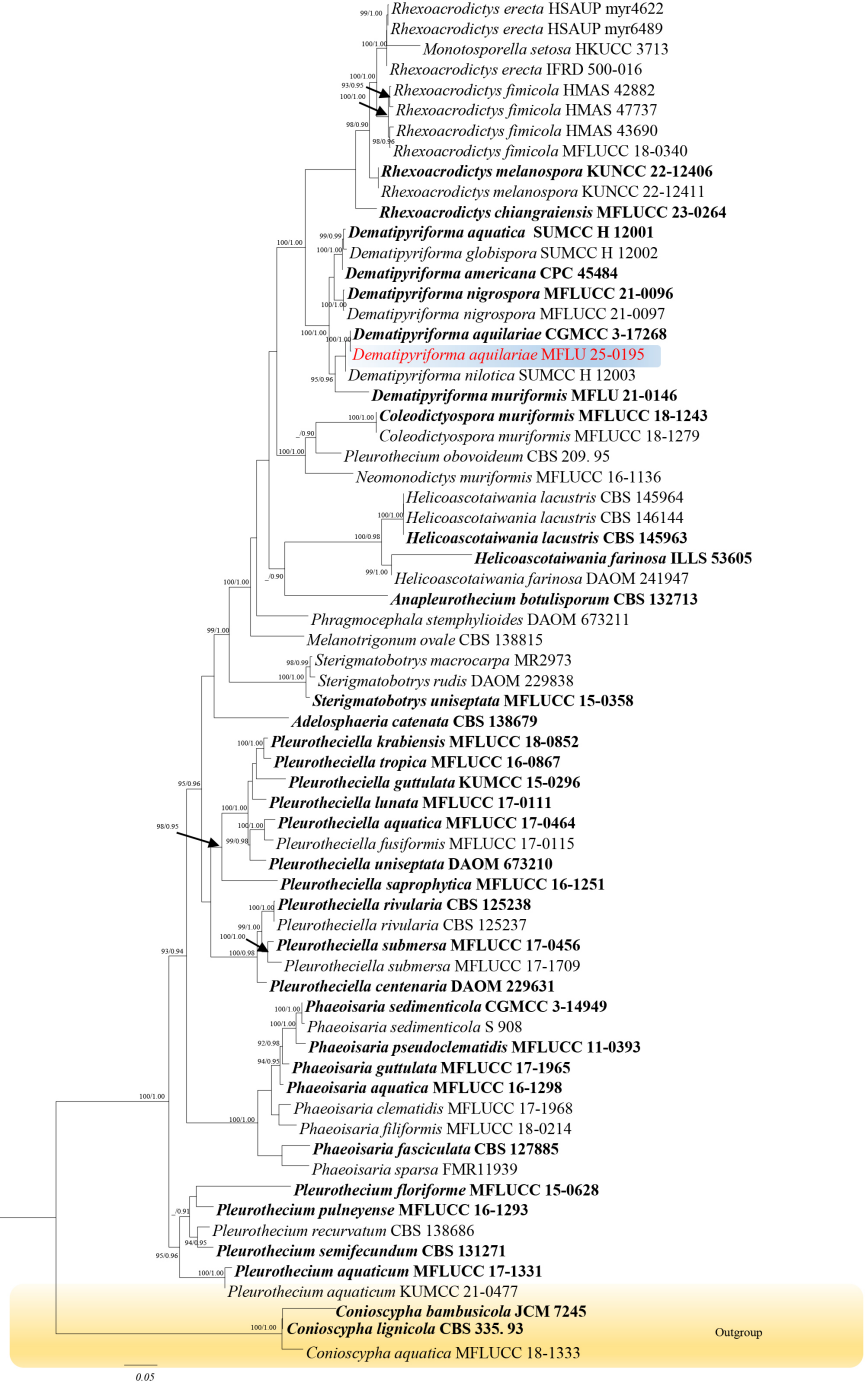
The best maximum likelihood tree with a final likelihood value of -27604.8853 for *Dematipyriforma* is presented. The phylogenetic tree constructed from maximum likelihood analysis based on the combined ITS, LSU, SSU and *rpb*2 sequence 67 strains are included in the combined sequence analysis, which comprised 4519 characters with gaps (ITS = 1–644, LSU = 645–1537, SSU = 1538–3481, *rpb*2 = 3482–4519). The tree topology of the maximum likelihood analysis is similar to the Bayesian analysis. The evolutionary model GTR+I+G is applied for all the genes. Bootstrap support values for ML equal or greater than 75% and Bayesian posterior probabilities greater than 0.90 are given near nodes respectively. The tree is rooted with *Conioscypha aquatica* (MFLUCC 18-1333), *C. bambusicola* (JMC 7245), and *C. lignicola* (CBS 335.93).

Phylogenetic analyses were based on combined gene datasets: ITS, LSU, SSU, and *rpb2* for *Dematipyriforma* (67 strains, 4519 characters, ITS = 1–644 bp, LSU = 645–1537 bp, SSU = 1538–3481 bp, *rpb2* = 3482–4519 bp, Fig. 1), showed *Dematipyriforma aquilariae* (MFLU 25-0195) clustering with *D. aquilariae* (CGMCC 3.17268) (Fig. 1).

**Figure 2.**
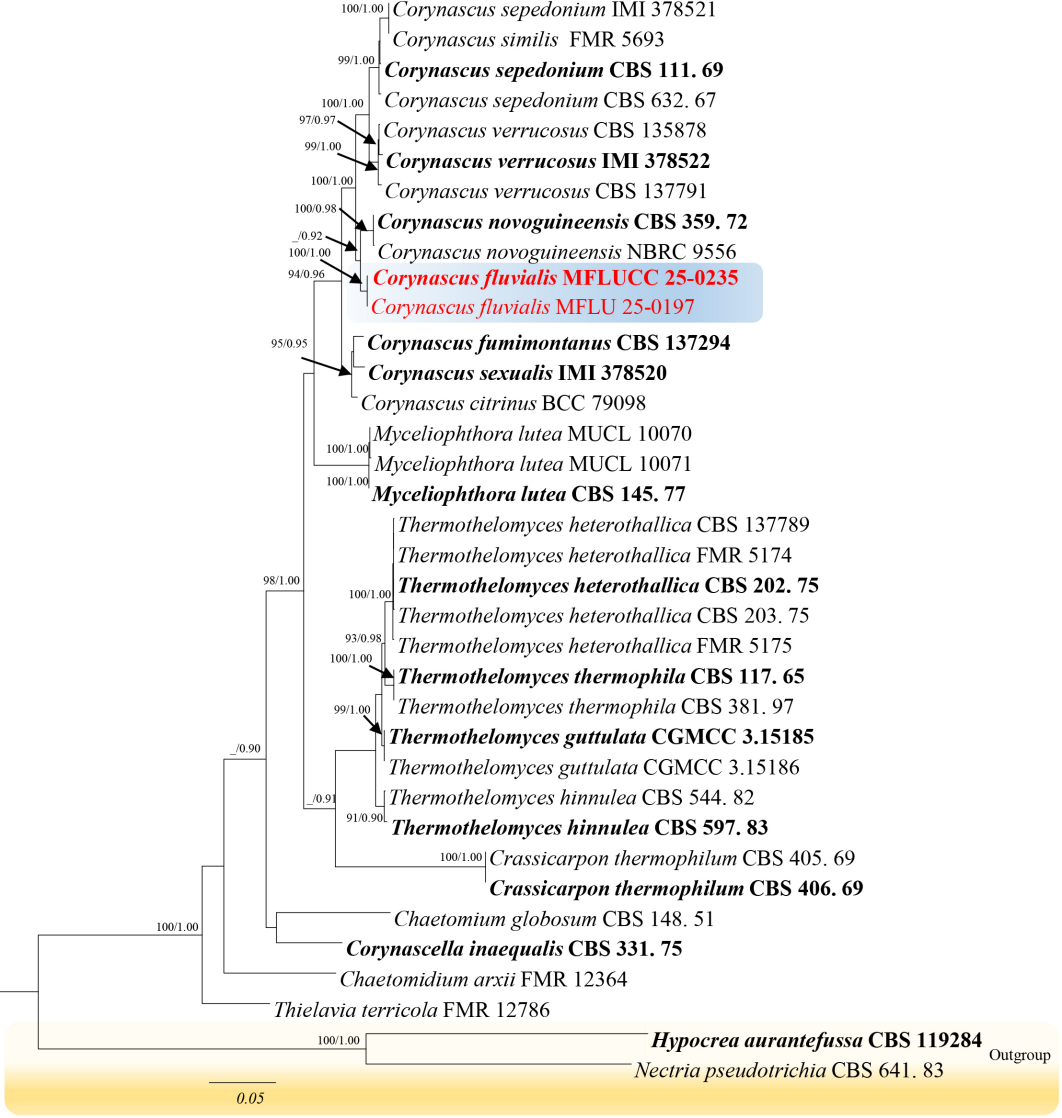
The best maximum likelihood tree with a final likelihood value of -12104.798 for *Corynascus* is presented. The phylogenetic tree constructed from maximum likelihood analysis based on the combined ITS, *tef1-α* and *rpb*2 sequence 35 strains are included in the combined sequence analysis, which comprised 2616 characters with gaps (ITS = 1–627, *tef1-α* = 628–1579, and *rpb*2 = 1580–2616). The tree topology of the maximum likelihood analysis is resembling to the Bayesian analysis. The evolutionary model GTR+I+G is applied for all the genes. Bootstrap support values for ML equal or greater than 90% and Bayesian posterior probabilities greater than 0.90 are given near nodes respectively. The tree is rooted with *Hypocrea aurantefussa* (CBS 119284) and *Nectria pseudotrichia* (CBS 641.83).

The multilocus phylogenetic analysis for *Corynascus* were implement using ITS, *tef1-α*, and *rpb2* included 35 strains (2616 characters, ITS = 1–627 bp, *tef1-α* = 628–1579 bp, *rpb2* = 1580–2616 bp), and the result showed that *C. fluvialis* (MFLUCC 25-0235) formed an independent lineage sister to *C. novoguineensis* (CBS 359.72, NBRC 9556) with moderate to strong support (89% ML, 0.90 BYPP, Fig. 2).

**Figure 3.**
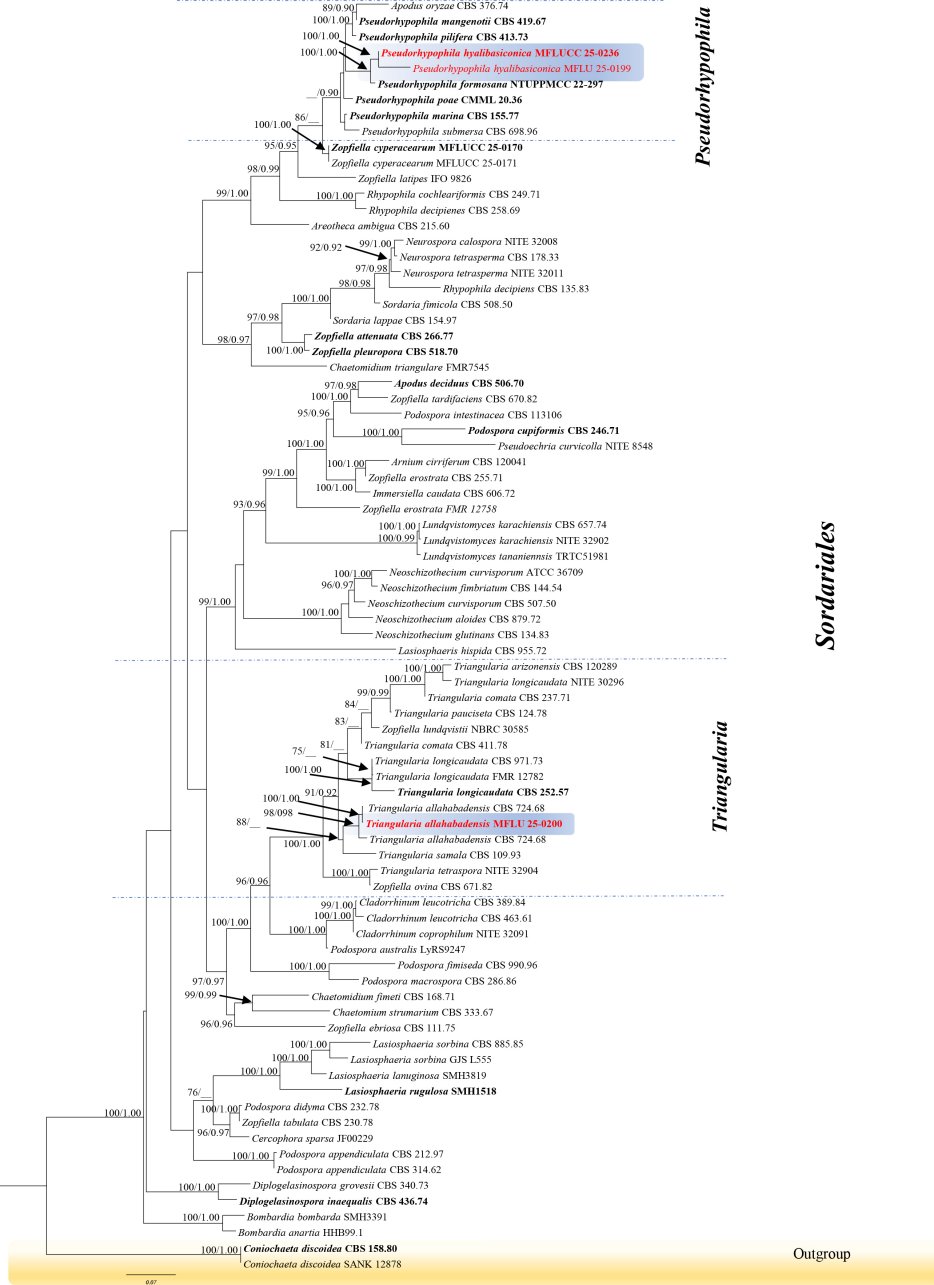
The best maximum likelihood tree with a final likelihood value of -42314.4773 is presented. Phylogenetic tree represents for *Pseudorhypophila hyalibasiconica* and *Triangularia allahabadensis*. The phylogenetic tree constructed from maximum likelihood analysis based on the combined LSU, ITS, *tub2* and *rpb2* sequence 81 strains are included in the combined sequence analysis, which comprised 3578 characters with gaps (LSU = 1–1112, ITS = 1113–1826, *tub2* = 1827–2371, *rpb2* = 2371–3578). The tree topology of the maximum likelihood analysis is similar to the Bayesian analysis. The evolutionary model GTR+I+G is applied for all the genes. Bootstrap support values for ML equal or greater than 75% and Bayesian posterior probabilities greater than 0.90 are given near nodes respectively. The tree is rooted with *Coniochaeta discoidea* (CBS 158.80 and SANK 12878).

The multilocus phylogenetic analysis for *Sordariales* was conducted emphasizing *Pseudorhypophila* and *Triangularia*. The phylogenetic tree constructed from the concatenated dataset of LSU, ITS, *tub2* and *rpb2* (81 strains, 3578 characters, LSU = 1–1112 bp, ITS = 1113–1826 bp, *tub2* = 1827–2371 bp, *rpb2* = 2371–3584 bp). Tree topologies from maximum likelihood analyses were similar to those from Bayesian analyses, with the evolutionary model GTR+I+G applied to all regions. *Triangularia allahabadensis* (MFLU 25-0200) formed a sister clade to *T. allahabadensis* (CBS 724.68) with strong statistical support (100% ML, 1.00 BYPP, Fig. 3), indicating close phylogenetic affinities to their sister strains. *Pseudorhypophila hyalibasiconica* (MFLUCC 25-0236) clustered with *Ps. formosana* (NTUPPMCC 22-297) with strong statistical support (100% ML, 1.00 BYPP, Fig. 3). These results support their recognition as new species within their respective genera.

### Taxonomy

The guidelines of Maharachchikumbura et al. (2021) were used when determining whether species were novel or new records.

#### *Pleurotheciales* Réblová & Seifert

***Pleurotheciaceae* Réblová & Seifert**

##### Dematipyriforma

Taxon classificationFungiPleurothecialesPleurotheciaceae

L.Y. Sun, Hai Y. Li, Xiang Sun & L.D. Guo

6F6A07A7-F713-5529-BFAC-D69629A01279

Index Fungorum: IF808026

Facesoffungi Number: FoF17783

###### Notes.

*Dematipyriforma* was introduced by Sun et al. (2017), under *Pleurotheciaceae*, *Pleurotheciales* in *Sordariomycetes*, and typified by *D. aquilariae*. The species have been reported from both aquatic and terrestrial environments (Sun et al. 2017; Bao et al. 2022). Endophytic and saprobic nutritional modes were reported from *Dematipyriforma* isolates from Asia (Bao et al. 2022). *Dematipyriforma* is characterized by immersed thick, light brown hyphae, monoblastic, intercalary, determinate, light brown and cylindrical conidiogenous cells. Conidiogenous cells are pyriform, smooth, thin-walled, transverse or longitudinal septate conidia. Conidia often possess single small basal cells connected to conidiogenous cells or to the hyphae (Sun et al. 2017; Bao et al. 2022).

##### Dematipyriforma aquilariae

Taxon classificationFungiPleurothecialesPleurotheciaceae

L.Y. Sun, Hai Y. Li, Xiang Sun & L.D. Guo

9FE7427F-9FE0-50F6-971B-30F5F4F746C3

Index Fungorum: IF842402

Facesoffungi Number: FoF12831

Fig. 4

###### Description.

***Saprobic*** on dead, decaying stems of *Typha* sp. **Sexual morph**: Undetermined. **Asexual morph**: Hyphomycetous. ***Colonies*** black, effuse, sporodochial. ***Mycelium*** 1.2–2.5 μm (x̄ = 2 μm, n = 30), immersed, smooth, subhyaline to pale brown, branched, septate, thin-walled. ***Chlamydospores*** 4–5 × 6.5–8.5 μm (x̄ = 4.5 × 7 μm, n = 30), intercalary or terminal, solitary, catenated, straight, or curved, brown to dark brown with a pale intermediate cell, smooth walls thickened with axial performative canals 1–8 transverse and occasionally 1 or more oblique or longitudinal septa, constricted at the septa. ***Conidiophores*** 2–6 μm wide (x̄ = 4.5 μm, n = 10), micronematous, mononematous, subhyaline to pale brown, smooth, straight to flexuous, septate. ***Conidiogenous cells*** 4.5–6 × 5.5–8 μm (x̄ = 5.5 × 7.8 μm, n = 30), holoblastic, integrated, intercalary, determinate, cylindrical, pale brown to brown with thin walls, conidial secession rhexolytic. ***Conidia*** 24–30 × 15–20 μm (x̄ = 26.5 × 17.5 μm, n = 30), pyriform or elongated pyriform, solitary, intercalary, smooth, thin-walled, muriform, rounded at the apex, pale grey olivaceous to pale brown, acropetal pigmentation during conidium formation.

**Figure 4.**
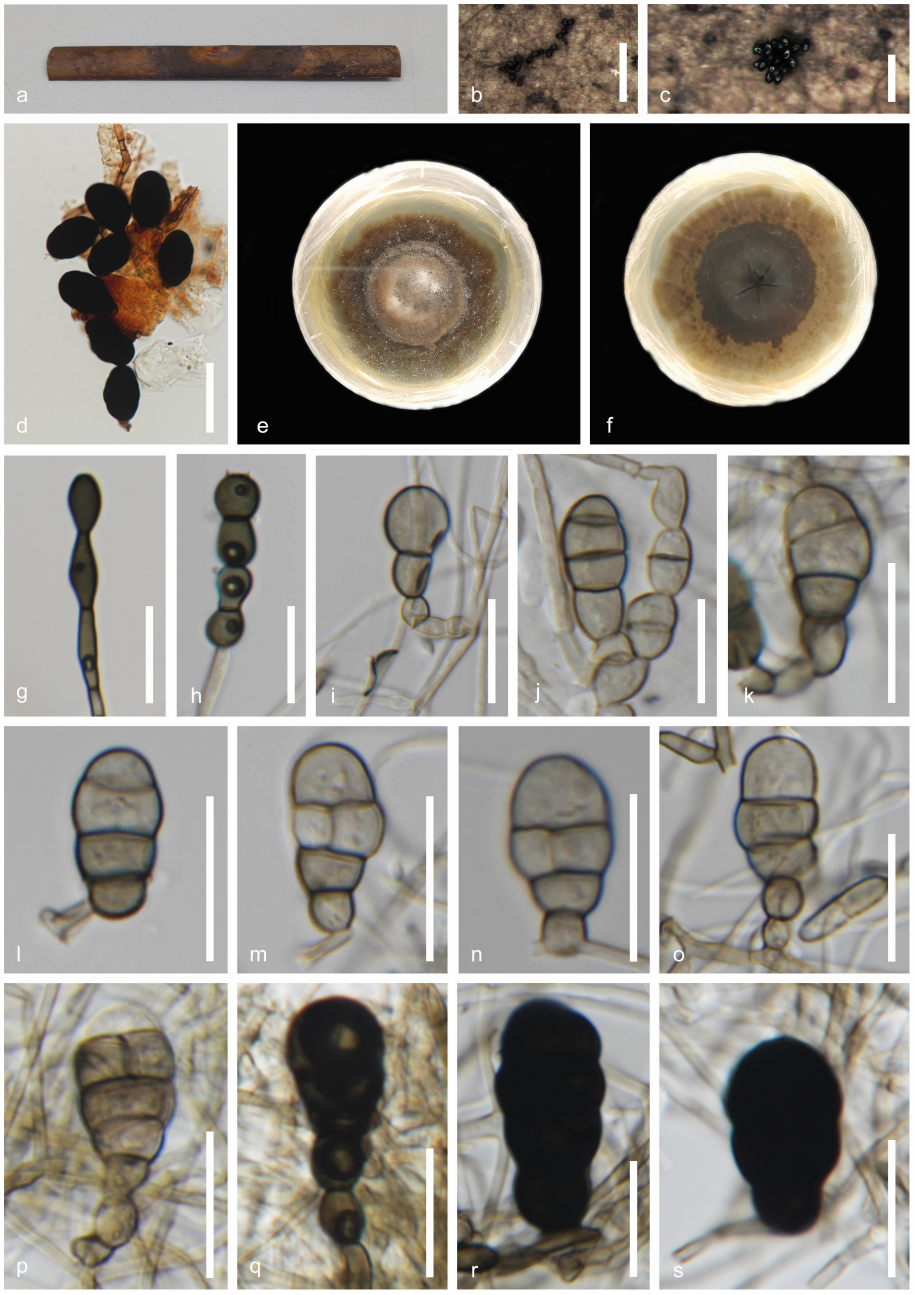
*Dematipyriforma aquilariae* (MFLU 25-0195). **a**. *Typha* sp. host material; **b, c**. Colonies on host surface; **d**. Squash mount of conidia; **e**. Culture on MEA above; **f**. Reverse; **g, h**. Chlamydospores; **i–s**. Conidial development. Scale bars: 500 µm (**b**); 250 µm (**c**); 50 µm (**d**); 20 µm (**g–s**).

###### Culture characteristics.

Conidia germinating on malt extract agar (MEA) within 24 h. Germ tube produced from the base of conidia. Colonies growing on MEA, reaching 40 mm in 4 weeks at 25 °C. Mycelia superficial, effuse to flat or undulate, from above olivaceous to greyish canter to the complete edge, from reverse greenish-black at the canter, light brown to black to hyaline edge.

###### Material examined.

Thailand • Prachuap Khiri Khan Province, Pran Buri District, Pran Buri Wetland, on decaying stems of *Typha* sp. (*Typhaceae*), 25 August 2023, Tharindu Bhagya, TB113 (MFLU 25-0195), living culture (MFLUCC 25-0242).

###### GenBank numbers.

ITS = PV759789, LSU = PV759788, SSU = PV759791, *rpb2* = PV779202.

###### Notes.

*Dematipyriforma aquilariae* (MFLU 25-0195) has a phylogenetic affinity with *D. aquilariae* (CGMCC 3.17268) and clusters with 100% ML and 1.00 BYPP support (Fig. 1). The new isolate agrees with the general description of *Dematipyriforma* by possessing monoblastic integrated conidiogenous cells and pyriform conidia with transverse, oblique or longitudinal septation (Sun et al. 2017; Bao et al. 2022). *Dematipyriforma aquilariae* (MFLU 25-0195) and *D. aquilariae* (CGMCC 3.17268) have rhexolytic conidial secession, smooth and thin-walled, pyriform or elongated pyriform, dark brown to black conidia with acropetal pigmentation (Fig. 4; Sun et al. 2017; Bao et al. 2022). Considering both phylogenetic and morphological evidence, we identify MFLU 25-0195 as a new isolate of *D. aquilariae*, and report this as a new host record for *Typha* sp., from freshwater coastal wetlands of Thailand.

#### *Sordariales* Chadef. ex D. Hawksw. & O.E. Erikss

***Chaetomiaceae* G. Winter**

##### Corynascus

Taxon classificationFungiSordarialesChaetomiaceae

Arx

22183E5D-F820-56CE-BF4C-8FF5671A271B

MB1257

Index Fungorum: IF1257

Facesoffungi Number: FoF17784

###### Notes.

*Corynascus* was introduced by von Arx (1973) and typified by *C. sepedonium*. It is characterized by sexual morphs that produce cleistothecial, soft, dark brown to black ascomata, comprising a peridium made of cells in *textura epidermoidea*, evanescent asci that bear eight ellipsoidal to fusiform ascospores in each ascus. Asexual morphs of the genus produce globose to subglobose, hyaline, thick-walled conidia (Marin-Felix et al. 2015; Crous et al. 2016). Members of the genus have a wide distribution including Asian, African continents, and European. *Corynascus* species are primarily saprobic and have been isolated from both terrestrial and aquatic habitats, particularly from decomposing plant material, as well as from soil. There is limited information on the evanescent nature of the asci and paraphyses in *Corynascus* species (Marin-Felix et al. 2015; Crous et al. 2016). Our investigation provides a detailed description of asci from the new isolate and reports the presence of paraphyses for the genus.

##### Corynascus fluvialis

Taxon classificationFungiSordarialesChaetomiaceae

Bhagya, Phukhams., K.D. Hyde & E.B.G. Jones sp. nov.

1EF44368-85C0-59C3-A955-63AB85B2329D

Index Fungorum: IF903990

Facesoffungi Number: FoF17785

Fig. 5

###### Etymology.

Based on the habitat of the host.

**Figure 5.**
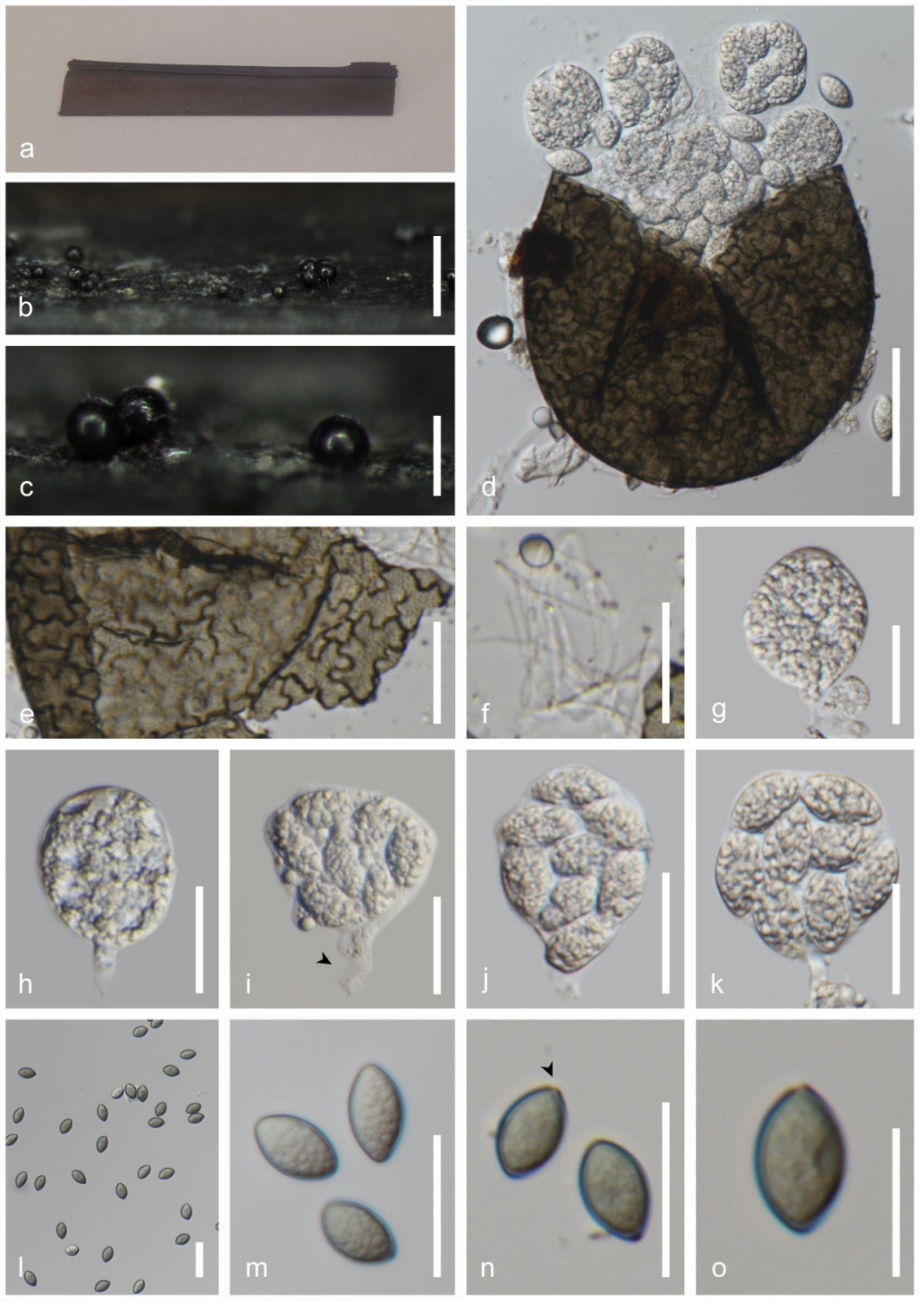
*Corynascus fluvialis* (MFLU 25-0196, holotype). **a**. *Carex* sp. host material; **b, c**. Superficial ascomata on the host tissue; **d**. Squash mount of ascoma in lactoglycerol; **e**. Peridium with visible textura epidermoidea cell type; **f**. Paraphysis mounted in lactoglycerol; **g–k**. Ascus development with angular pedicel (arrowed); **l–o**. Ascospores with single apical germ pore (arrowed). Scale bars: 250 µm (**d**); 100 µm (**c**); 50 µm (**d**); 20 µm (**e–n**); 10 µm (**o**).

###### Holotype.

MFLU 25-0196.

###### Description.

***Saprobic*** on dead, moist, decaying stems of *Carex* sp. **Sexual morph: *Ascomata*** 110–150 (x̄ = 124 μm, n = 5), cleistothecial, scattered, loosely aggregated or gregarious, superficial on host surface, dark brown, to black or olivaceous, globose to subglobose, smooth, and coriaceous. ***Peridium*** 1–3 μm (x̄ = 1.5 μm, n = 5) composed of olivaceous brown cells in *textura epidermoidea*. ***Paraphyses*** 12–25 × 1–2.5 μm (x̄ = 18.5 × 1.5 μm, n = 5), filiform, aseptate, hyaline, shorter than the asci. ***Asci*** 25–38 × 22–35 μm (x̄ = 35 × 28 μm, n = 10), 8-spored, unitunicate, globose or subglobose to pyriform or broadly clavate, thin-walled, caudate with short cylindrical to angular pedicel, evanescent. ***Ascospores*** 12–18 × 6–10 μm (x̄ = 14.5 × 8.5 μm, n = 30), overlapping, biseriate to triseriate or variably arranged, ellipsoidal or broadly fusiform, straight, aseptate, hyaline when immature, olivaceous-green when mature, thick, smooth-walled, pointed towered end, with one germ pore at apex. **Asexual morph**: undetermined.

###### Culture characteristics.

Ascospore germinating on malt extract agar (MEA) within 72 h. Germ tubes are produced from the apical ends of the ascospores. Colonies growing on MEA, reaching 20 mm in 4 weeks at 25 °C. Mycelia superficial, umbonate with a complete edge, from above light grey at the canter, off white to hyaline towards edge, from reverse dark greenish ash from the canter surrounded with off-white ring at the edge.

###### Material examined.

Thailand • Prachuap Khiri Khan Province, Pran Buri District, Pranburi river, on decaying leaf of *Carex* sp. (*Cyperaceae*), 25 August 2023, Tharindu Bhagya, TB130 (MFLU25-0196, holotype); ex-type living culture (MFLUCC 25-0235); *ibid*., TB130B (MFLU25-0197).

###### GenBank numbers.

MFLU 25-0196: ITS = PV764283, *rpb2* = PV799940, *tef1-α* = PV799942; MFLU 25-0197: ITS = PV764282, *rpb2* = PV799941.

###### Notes.

The newly isolated strain (MFLUCC 25-0235) clustered with *Corynascus novoguineensis* (CBS 359.72 and NBRC 9556) with 89% ML and 0.90 BYPP support (Fig. 2). Strain MFLUCC 25-0235 shares common characteristics with *Corynascus*, including cleistothecial ascomata, a peridium composed of cells in *textura epidermoidea*, and fusiform ascospores (Marin-Felix et al. 2015; Crous et al. 2016). Phylogenetically, the new strain differs from the type strain of *C. novoguineensis* (CBS 359.72) by 2.8% in ITS, 3.2% in *tef1-α*, and 4.4% in *rpb2* in pairwise comparisons, excluding gaps. Morphologically, the new isolate is distinguishable from *C. novoguineensis* (CBS 359.72 and NBRC 9556) by possessing a smooth ascomatal wall, asci with cylindrical to angular pedicel and hyaline immature ascospores that change to olivaceous-green at maturity (Fig. 5). In comparison, *C. novoguineensis* (CBS 359.72 and NBRC 9556) develops a verrucous ascomatal wall and pinkish immature ascospores. The presence of hyaline, filiform paraphyses makes the new isolate unique among related taxa in the genus *Corynascus* (Marin-Felix et al. 2015; Crous et al. 2016). Considering the available morphological and phylogenetic evidence, we introduce *C. fluvialis* (MFLUCC 25-0235) as a new species from freshwater wetlands in Thailand.

#### *Naviculisporaceae* Y. Marin & Stchigel

***Schizotheciaceae* Y. Marin & Stchigel**

##### Pseudorhypophila

Taxon classificationFungiSordarialesNaviculisporaceae

Y. Marín & Stchigel, (2021)

E288252E-BB10-5902-958D-9F86E6934A18

Index Fungorum: IF838466

Facesoffungi Number: FoF14642

###### Notes.

*Pseudorhypophila* was proposed by Marín and Stchigel in 2021 by synonymising *Triangularia mangenotii* under *Pseudorhypophila* as *P. mangenotii* (CBS 419.97) (Harms et al. 2021). Members of *Pseudorhypophila* are characterized by superficial or immersed, globose, subglobose, ovate to pyriform, black or dark brown ascomata covered with flexuous hairs, clavate to cylindrical asci that bear either 4 or 8 ascospores and occasionally possess inconspicuous apical ring at each ascus. Ascospores are predominantly 2-celled, upper cells are usually larger, olivaceous brown to dark brown, ovoid to limoniform and truncate at the base. Some species have subapical germ pores or with distinct apical appendages. Lower cells of the ascospores are hyaline, pale olivaceous brown or pale brown, cylindrical and straight or curved, or hemispherical. Asexual morphs of *Pseudorhypophila* exhibit spherical to ovate to elongate, hyaline conidia that originate directly from the vegetative hyphae (Harms et al. 2021).

##### Pseudorhypophila hyalibasiconica

Taxon classificationFungiSordarialesNaviculisporaceae

Bhagya, Phukhams., K.D. Hyde & E.B.G. Jones sp. nov.

D27CBBC6-48F6-58E7-B2B2-0CE616D57462

Index Fungorum: IF903989

Facesoffungi Number: FoF17786

Fig. 6

###### Etymology.

Based on the ascospore morphology.

**Figure 6.**
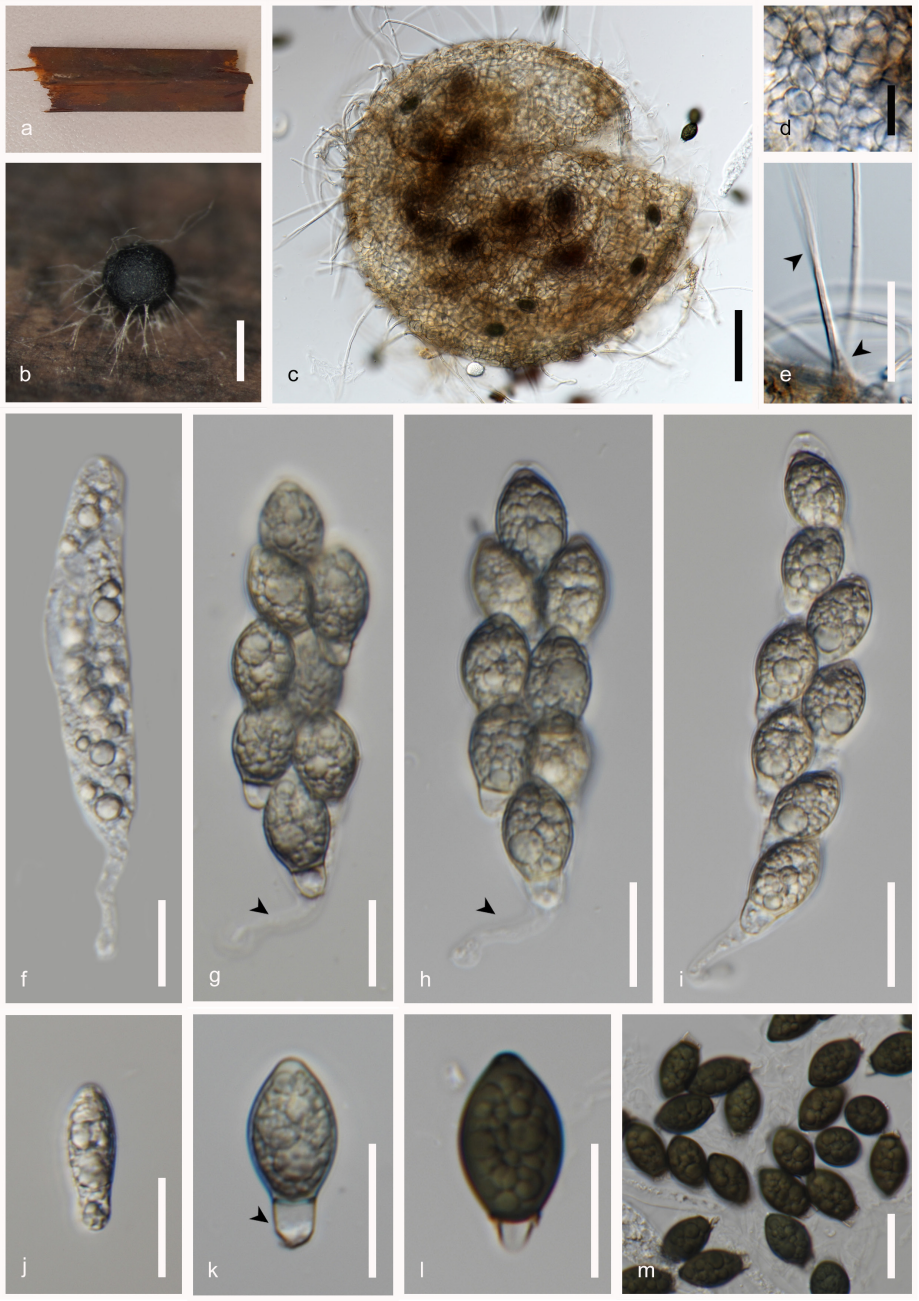
*Pseudorhypophila hyalibasiconica* (MFLU 25-0198, holotype). **a**. *Carex* sp. host material; **b**. Black spherical cleistothecial ascoma on the host; **c**. Squashed mount of ascoma; **d**. Surface of peridium; **e**. Ascomatal hairs originating directly from peridium cells (arrowed); **f–i**. Asci with long curved pedicel (arrowed); **j–m**. Ascospores with hyaline basal cell (arrowed). Scale bars: 500 µm (**b**); 50 µm (**c**); 20 µm (**d–m**).

###### Holotype.

MFLU 25-0198.

###### Description.

***Saprobic*** on dead and decaying leaf of a *Carex* sp., appearing as black spherical globes loosely attached to the host tissue. **Sexual morph: *Ascomata*** 300–450 μm (x̄ = 320 μm, n = 5) cleistothecial, gregarious in aggregation, scattered on the host, superficial on host surface, globose, black, smooth-walled, non-ostiolate, covered with hyaline to light brown flexuous ascomatal hairs. ***Peridium*** 15–20 μm wide (x̄ = 18 μm, n = 10), relatively thin, single–layer comprising black to light brown cells of *textura angularis* and *textura intricata*. ***Paraphyses*** not observed. ***Asci*** 90–150 × 20–28 μm (x̄ = 112 × 24 μm), 8-spored, unitunicate, highly evanescent, cylindrical to subcylindrical or clavate, long pedicellate with nonamyloid (J-) apices. ***Ascospores*** 24–30 × 12–14 μm (x̄ = 25.5 × 13.5 μm, n = 20), uniseriate or overlapping biseriate, upper cell, 17–22 × 11–14 μm (x̄ = 19.5 × 12.5 μm, n = 20), fusiform, narrowing towards apex, apiculate, ovoid to limoniform, hyaline when young, greenish-brown to dark brown at maturity, aseptate, smooth-walled; basal cell 5–7 × 4–6 μm (x̄ = 5.5 × 5.2 μm, n = 10), limoniform when young, conical when at maturity, deliquescing, pedicel-like, hyaline, originating at the base, narrowing to blunt base. **Asexual morph**: Undetermined.

###### Material examined.

Thailand • Prachuap Khiri Khan Province, Pran Buri District, Pranburi River, on decaying leaf of *Carex* sp. (*Cyperaceae*), 25 August 2023, Tharindu Bhagya, TB125 (MFLU 25-0198, holotype); ex-type living culture, MFLUCC 25-0236; *ibid*., TB125B (MFLU 25-0199, isotype).

###### GenBank numbers.

MFLU 25-0198: ITS = PV764279, LSU = PV764280, *rpb2* = PV786267, *tub2* = PV799943; MFLU 25-0199: ITS = PV764278, LSU = PV764281, *rpb2* = PV786268.

###### Notes.

*Pseudorhypophila hyalibasiconica* (MFLUCC 25-0236) clustered with *Ps. formosana* (NTUPPMCC 22-297) with 100% ML and 1.00 BYPP statistical support (Fig. 3). The new isolate agrees with the general description of *Pseudorhypophila* by possessing globose, subglobose ascomata with flexuous ascomatal hairs, unitunicate asci with inconspicuous apical rings and 2-celled ascospore with olivaceous brown to dark brown, ovoid to limoniform upper cells and hyaline, pale olivaceous smaller lower cell (Fig. 6; Harms et al. 2021). *Pseudorhypophila hyalibasiconica* (MFLUCC 25-0236) is phylogenetically distinct from *Ps. formosana* (NTUPPMCC 22-297) with 3.28% in ITS, 1.01% in LSU, 7.32% in *tub2* and 8.19% for the *rpb2* in base pairs including gaps. *Pseudorhypophila mangenotii* differs from other species based on the presence of an ostiole to the ascomata, while *P. pilifera* and *P. marina* are distinguishable from each other based on the shape of the ascospores basal cell (Harms et al. 2021). *Pseudorhypophila hyalibasiconica* (MFLUCC 25-0236) is distinguishable from closely related species, *Ps. formosana* (NTUPPMCC 22-297), and *Ps. mangenotii* (CBS 419.97) by possessing non-ostiolate, cleistothecial ascomata. It further distinguishes itself from *Ps. pilifera* (CBS 413.73) by ovoid to limoniform upper cells and conical lower cell at maturity. *Pseudorhypophila* currently comprises five species, with this study introducing *Ps. hyalibasiconica* as the sixth species from Thailand (Harms et al. 2021; Cheng et al. 2025).

##### Triangularia

Taxon classificationFungiSordarialesNaviculisporaceae

Boedijn, Ann. Mycol. 32: 302 (1934)

92191F64-9061-577A-9B37-B34D3A6AD27B

Index Fungorum: IF5534

Facesoffungi Number: FoF13547

###### Notes.

*Triangularia* was introduced by Boedijn (1934), typified by *T. bambusae*. According to the current classification, the genus resides under *Schizotheciaceae*, *Sordariales*, *Sordariomycetidae* (Wang et al. 2019; Marin-Felix et al. 2020; Hyde et al. 2024). Members of the genus *Triangularia* are distinguishable from closely related taxa by possessing cylindrical to clavate asci with a prominent apical ring, predominately 1-sepate or aseptate, smooth ascospores, which consist of relatively large pigmented, conical or triangular upper cell and hyaline to sub-hyaline and triangular to hemispherical lower cell. The gelatinous appendages are yet to be observed in the genus (Wang et al. 2019; Marin-Felix et al. 2020).

##### Triangularia allahabadensis

Taxon classificationFungiNaviculisporaceae

(M.P. Srivast., Tandon, Bhargava & A.K. Ghosh) X. Wei Wang & Houbraken

6B7CA2FF-AE8A-5A20-AA3E-310C07B433C4

Index Fungorum: IF829884

Facesoffungi Number: FoF17787

Fig. 7

###### Description.

***Saprobic*** on dead and decaying leaf of a *Carex* sp., appearing as black spherical globes loosely attached to the host tissue. **Sexual morph: *Ascomata*** 380–520 × 360–570 μm (x̄ = 410 × 440 μm, n = 5) scattered on the host, superficial, perithecial, subglobose or ampulliform, carbonaceous, papillated, black, smooth-walled, with short ostiole, covered with hyaline to light brown ascomatal hairs. ***Peridium*** carbonaceous, black to greenish-brown cells of *textura angularis*. ***Paraphyses*** 120–180 × 2–4 μm (x̄ = 142 × 2.5 μm) smooth-walled, hyphae-like and septate. ***Asci*** 90–160 × 15–22 μm (x̄ = 135 × 19 μm), 8-spored, fasciculate, unitunicate, evanescent, cylindrical to subcylindrical or clavate, short pedicellate with a nonamyloid apex. ***Ascospores*** upper cell, 24–32 × 8–12 μm (x̄ = 28.5 × 9.5 μm, n = 20), fusiform or semi-spheroid, narrowing towards the apices, apiculate, hyaline when young, greenish-brown to dark brown at maturity, aseptate, coarse at maturity; immature spores process a basal cell that is evanescent and disappears at spore maturity; basal cell 9–16 × 2–4 μm (x̄ = 13.2 × 3.5 μm, n = 10), semi-curricular, pedicel-like, hyaline, originating at the base, narrowing to the pointed base. **Asexual morph**: Undetermined.

**Figure 7.**
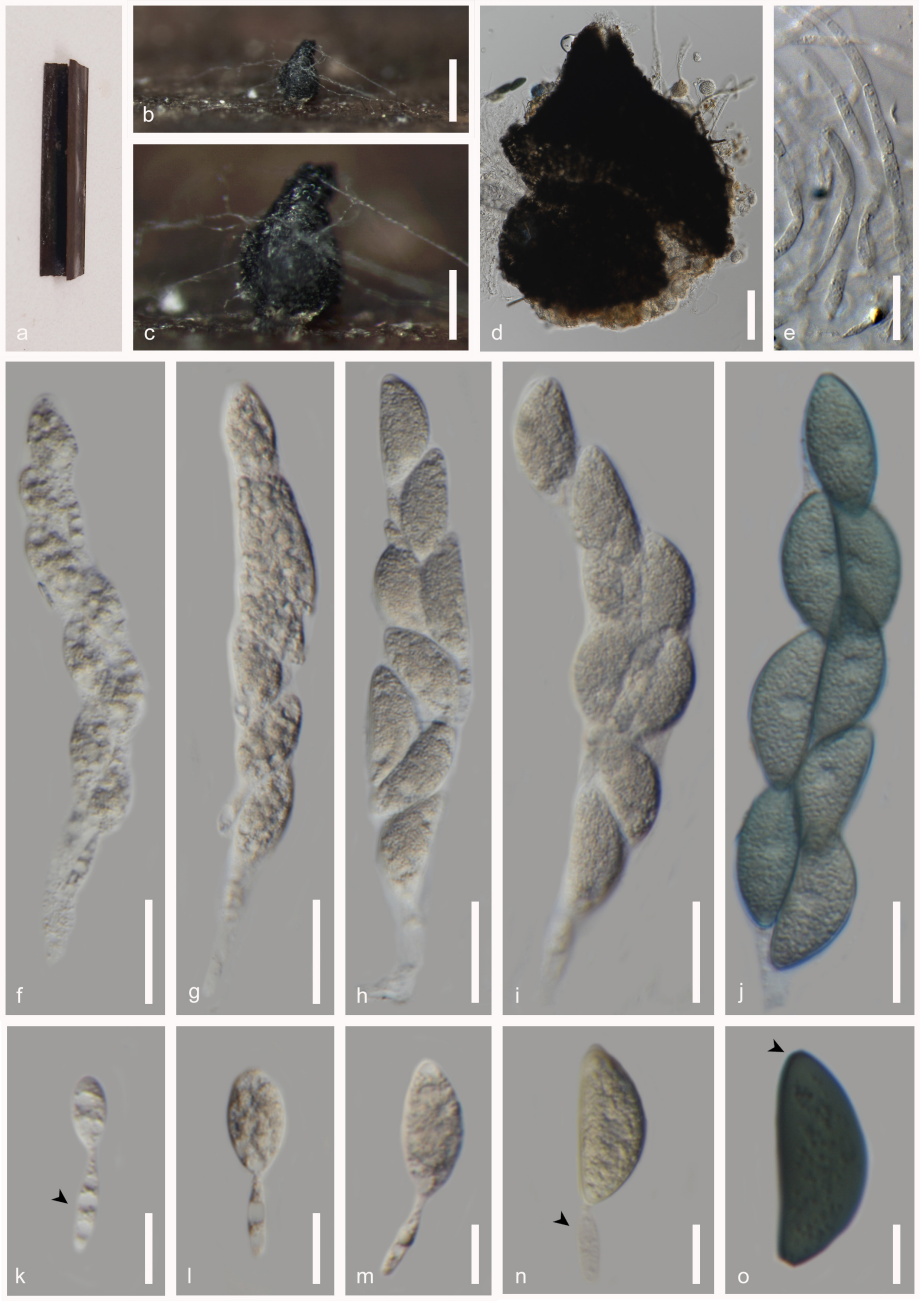
*Triangularia allahabadensis* (MFLU 25-0200). **a**. *Carex* sp. host material; **b, c**. Black ascoma on the host; **d**. Squashed mount of ascoma; **e**. Hyphae-like paraphyses; **f–j**. Immature and mature asci; **j–m**. Ascospores with hyaline base cell disappearing at spore maturity (arrowed). Scale bars: 500 µm (**b**); 250 µm (**c**); 50 µm (**d**); 20 µm (**e–j**); 10 µm (**k–o**).

###### Material examined.

Thailand • Prachuap Khiri Khan Province, Pran Buri District, Pranburi River, on decaying leaf of *Carex* sp., (*Cyperaceae*), 25 August 2023, Tharindu Bhagya, TB127 (MFLU 25-0200).

###### GenBank numbers.

ITS = PV759786, LSU = PV759787, *rpb2* = PV779203, *tub2* = PV786266.

###### Note.

Our isolate MFLU 25-0200, clustered basal to *Triangularia allahabadensis* CBS 724.68 with 100% ML and 1.00 BYPP bootstrap support (Fig. 3). MFLU 25-0200 has general morphological characters of *Triangularia* in producing ostiolate and ovoid, obpyriform to ampulliform, globose ascomata, cylindrical to elongated or clavate asci, septate ascospores with larger pigmented, conical or triangular upper cell and triangular to hemispherical, paler lower cell (Wang et al. 2019; Marin-Felix et al. 2020). The new isolate collected from *Carex* species, shares significant morphological similarities with the sister taxon, *T. allahabadensis* (CBS 724.68) in having fasciculate, cylindrical, subcylindrical or clavate asci that lack a conspicuous apical ring, and fusiform, dark to greenish-brown ascospores with evanescent smaller basal cells (Fig. 7; Wang et al. 2019; Marin-Felix et al. 2020). Considering available morphological and phylogenetic evidence we recognise MFLU 25-0200 as a new strain of *Triangularia allahabadensis*. Therefore, we document the first report of *T. allahabadensis* from *Carex* species collected in Thailand.

## Discussion

*Sordariomycetes* are associated with a wide range of habitats, including wetland marshes (Luo et al. 2019; Hyde et al. 2020; Marin-Felix et al. 2020). The isolates discussed in this study originated from freshwater coastal wetlands in Thailand subject to annual rainfall and rivers. According to the ecology of these environments, water salinity fluctuated between 0.0% and 0.2%, effectively classifying these study sites as freshwater ecosystems.

*Dematipyriforma aquilariae* (MFLU 25-0195) was recorded from *Typha (Typhaceae)* in the Pran Buri wetland as a new host and geographical record. Members of this genus are predominantly found as saprobes on submerged freshwater wood in Asia and Africa. For example, *Dematipyriforma aquatica* (SUMCC H-12001) and *D. globispora* (SUMCC H-12002) were isolated from submerged wood in Egypt (Abdel-Aziz and Bakhit 2023), while *D. muriformis* (MFLU 21-0146) was recorded on submerged wood in a freshwater stream in Thailand (Sun et al. 2017; Bao et al. 2022; Yang et al. 2023). The most recent species, *Dematipyriforma americana* (CBS H-25310), was introduced by Visagie et al. (2024) and isolated from a basement wall in the United States. These findings emphasize the affinity of *Dematipyriforma* species for aquatic environments and highlight their adaptability to withstand harsh and dynamic environmental conditions. Our isolate was found close to the water-air interface of a lentic water body. Based on this, the authors speculate that the sporulation of *D. aquilariae* may be triggered by a drop in water level, exposure to sunlight, and dry conditions, utilizing both water and wind for conidial dispersal. This suggests that *D. aquilariae* may exhibit characteristics similar to those of amphibious or aero-aquatic hyphomycetes, such as, *Dematiosporium*, and *Rhexoacrodictys* (Luo et al. 2019; Bao et al. 2023; Win et al. 2024).

The order *Sordariales* in *Sordariomycetes* includes several families, such as *Lasiosphaeriaceae*, *Podosporaceae*, and *Naviculisporaceae*, which comprise members associated with aquatic environments (Maharachchikumbura et al. 2016; Phookamsak et al. 2019; Hyde et al. 2020; Chen et al. 2023). The delineation of genera within these families, particularly in *Lasiosphaeriaceae*, has traditionally relied on ascospore characteristics, often resulting in polyphyletic genera (Harms et al. 2021). Modern taxonomic approaches, combining morphological examinations with multi-locus phylogenetic analyses, have significantly contributed to resolving their controversial and uncertain taxonomic placements. For example, a study using ITS, LSU, *rpb2*, and *tub2* loci revealed that the type strain of *Triangularia* (= *T. mangenotii*) clusters within *Naviculisporaceae*, distinct from other related taxa in *Podosporaceae*. Thus, *Pseudorhypophila* was introduced for *Triangularia mangenotii* (Harms et al. 2021). *Pseudorhypophila* exhibits close morphological and evolutionary relationships with *Rhypophila*. However, *Rhypophila* can be distinguished from *Pseudorhypophila* by their elongated, tuberculate projections on ostioles and ascospores with lower cells that are either as long as or longer than the upper cells (Harms et al. 2021).

Harms et al. (2021) transferred *Zopfiella marina* and *Z. pilifera* to *Pseudorhypophila* based on multigene phylogenetic evidence and chemotaxonomic analyses. Observations and recent studies suggest that relying primarily on ascospore shape for genus delineation is phylogenetically inaccurate, particularly in families such as *Lasiosphaeriaceae*, *Podosporaceae*, *Naviculisporaceae*, and *Schizotheciaceae* (Hyde et al. 2020; Harms et al. 2021; Chen et al. 2023). Current taxonomic understanding of *Pseudorhypophila* highlights that the shape of ascospore cells and the presence or absence of an ostiole hold significant taxonomic importance in delineating species in an already established genus (Harms et al. 2021). Further in-depth phylogenetic analyses, coupled with morphological and chemotaxonomic studies, are recommended to clarify the uncertain taxonomic placements associated with neighbouring families related to *Naviculisporaceae*.

Another closely related species discussed in this manuscript is *Triangularia allahabadensis* (MFLU 25-0200), a species isolated from decomposing submerged *Carex* material in the Pran Buri River. *Triangularia allahabadensis* was originally isolated from *Carica papaya* flowers collected from India (Srivastava et al. 1966). The species initially described as *Sordaria allahabadensis*, was later transferred to *Triangularia* based on its morphological characteristics (Wang et al. 2019). Previous studies have treated *Sordaria allahabadensis* as a synonym of *Podospora austroamericana* (Marin-Felix et al. 2020). However, distinct morphological features, including a phialophora-like morph and clavate, persistent, broader primary appendages on the ascospores, differentiate it from *Triangularia allahabadensis* (Mirza and Cain 1969; Guarro et al. 1991). Wang et al. (2019) and Marin-Felix et al. (2020) highlighted the importance of conducting further in-depth studies to clarify the relationship between *Podospora austroamericana*, *Sordaria allahabadensis*, and *Triangularia allahabadensis*. We concur with these authors and emphasize that these species demand additional investigations combined with morphology, multigene phylogeny and chemotaxonomy.

Fungi under the families *Chaetomiaceae*, *Naviculisporaceae*, *Pleurotheciaceae*, and *Schizotheciaceae* are known to exhibit different nutritional or life modes, including saprobes, pathogens, and endophytes (Luo et al. 2019; Huang et al. 2021). *Dematipyriforma aquilariae* (CGMCC 3.17268), the type species of *Dematipyriforma* was recollected in this study (MFLU 25-0195), as a saprobe. *Dematipyriforma aquilariae* was originally introduced and described as an endophyte in *Aquilaria crassna* from Laos by Sun et al. (2017). Members of *Triangularia* and *Corynascus* include soil-associated and endophytic species. For example, *T. allahabadensis* has been reported from soil and identified as a Dark Septate Endophyte (DSE) associated with *Smilax regelii* in a desert region of Northwest China (Zuo et al. 2022). The most recent species in the genus *Triangularia* is *T. manubriata* (KNUF-21-020), a strain introduced as an edaphic taxon by Lim et al. (2024). Species within the genus *Corynascus* have also been reported to inhabit soil, such as *C. fumimontanus* isolated from forest soil in Tennessee (Marin-Felix et al. 2015). *Pseudorhypophila marina* (basionym: *Zopfiella marina* Furuya & Udagawa, 1975) has also been reported as an edaphic taxon, isolated from marine mud (Harms et al. 2021). Additionally, *P. mangenotii* (Egy7-Gray) was isolated as an endophyte from the roots of tomato plants in Egypt (El-Debaiky and El-Sayed 2023). Other related taxa, including members of *Zopfiella* and *Podospora*, are known to include both endophytic and edaphic organisms (Hyde et al. 2020; Marin-Felix et al. 2020).

Considering this evidence, the authors hypothesize that the taxa isolated and described in this study have the potential to inhabit hosts as endophytes and may later switch to a saprobic nutritional mode following the host’s death (Bhunjun et al. 2024). Initial fungal infection may occur through the root system from the soil. Further investigations are necessary to develop a clearer understanding of the host-fungal relationships associated with wetland-dwelling *Poales* in Central Thailand.

## Supplementary Material

XML Treatment for Dematipyriforma

XML Treatment for Dematipyriforma aquilariae

XML Treatment for Corynascus

XML Treatment for Corynascus fluvialis

XML Treatment for Pseudorhypophila

XML Treatment for Pseudorhypophila hyalibasiconica

XML Treatment for Triangularia

XML Treatment for Triangularia allahabadensis

## Additional information

### Use of AI

No use of AI was reported.

### Author ORCIDs

Amuhenage T. Bhagya https://orcid.org/0009-0002-3988-298X

Chayanard Phukhamsakda https://orcid.org/0000-0002-1033-937X

Pattana Kakumyan https://orcid.org/0000-0001-8281-1082

Ali H. Bahkali https://orcid.org/0000-0003-3936-0436

E.B. Gareth Jones https://orcid.org/0000-0002-7286-5471

Kevin D. Hyde https://orcid.org/0000-0002-2191-0762

### Data availability

All of the data that support the findings of this study are available in the main text, cultures and herbarium materials are deposited in MeaFah Luang University Culture collection and herbarium respectively.
